# Supplementary material for: Structural and electronic signatures of strain-tunable marginally twisted bilayer graphene
Source: Natl Sci Rev. 2025 Dec 11;13(4):nwaf568. doi: 10.1093/nsr/nwaf568 (PMC12902690; doi:10.1093/nsr/nwaf568)
Supplement: nwaf568_Supplemental_File [file nwaf568_supplemental_file.pdf]

Supplemental Material

Structural and electronic signatures of strain-tunable marginally twisted bilayer graphene

Pei Ouyang[1,2], Jiawei Yu[1,2], Qian Li[1,2], Guihao Jia[1,2], Yuyang Wang[1,2], Kebin Xiao[1,2], Hongyun Zhang[1,2], Zhiqiang Hu[1,2], Pierre A. Pantaleón[3], Zhen Zhan[3,*], Shuyun Zhou[1,2], Francisco Guinea[3,4], Qi-Kun Xue[1,2,5,6,7,*], and Wei Li[1,2,7,*]

[1]*State Key Laboratory of Low-Dimensional Quantum Physics,*
Department of Physics, Tsinghua University, Beijing 100084, China

[2]*Frontier Science Center for Quantum Information, Beijing 100084, China*

[3]*Imdea Nanoscience, Faraday 9, 28015 Madrid, Spain*

[4]*Donostia International Physics Center, Pasco Manuel de Lardizábal 4, 20018 San Sebastián, Spain*

[5]*Beijing Academy of Quantum Information Sciences, Beijing 100193, China*

[6]*Southern University of Science and Technology, Shenzhen 518055, China*

[7]*Hefei National Laboratory, Hefei 230088, China*

**To whom correspondence should be addressed:* zhen.zhan@imdea.org; qkxue@mail.tsinghua.edu.cn; weili83@tsinghua.edu.cn

I. DOMAIN WALL TYPES AND ALTERNATIVE LDOS EXPLANATIONS IN DW AND AB REGIONS

1, Shear and tensile domain wall

Fig. S1 shows two types of AB-BA domain boundaries in bilayer graphene: shear DW and tensile DW. The shear DW follows an armchair orientation, while the tensile DW aligns along the zigzag direction. The electronic properties of these DWs are strongly influenced by both the boundary orientation and the atomic configuration within the DW [1–3]. The width of the DWs in the STM topography is shown in Fig. S2. The DW widths are measured to be approximately 10 nm, in agreement with previous reports [4,5].

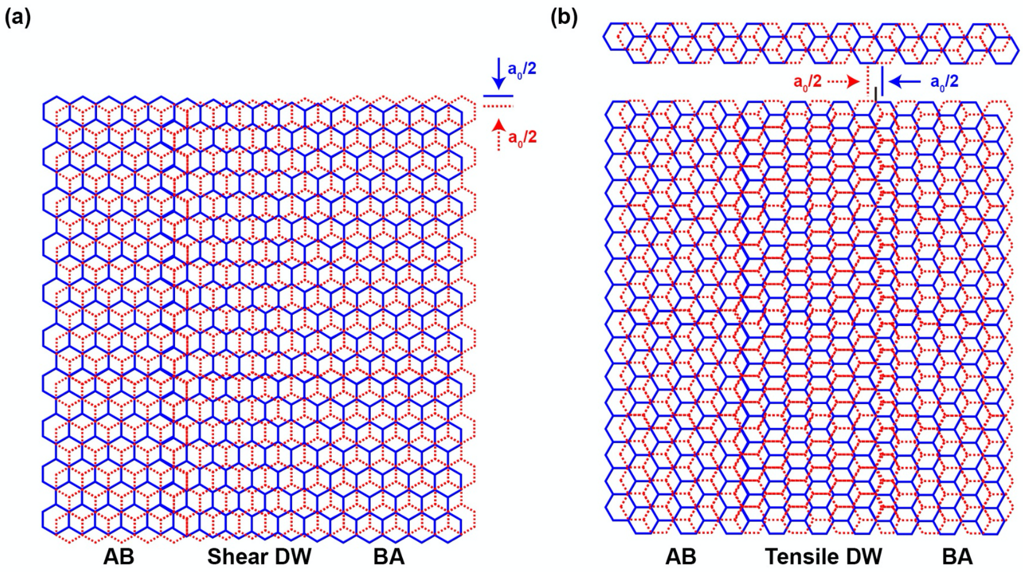

Figure S1. **Schematic of shear DW and tensile DW.** (**a**), Shear DW. (**b**), Tensile DW.

2, Alternative LDOS Explanation in AB region

Although electron confinement can give rise to discrete peaks in the local density of states (LDOS), energy considerations rule out this mechanism in our case. As shown in Fig. 2(c), five discrete LDOS peaks appear between -100 meV and 0 meV, with energy spacings ranging from 15 to 30 meV. If we model the AB region as a quantum well with a width approximated by the moiré period (~300 nm), the expected energy level spacing can be estimated using the expression $\Delta E \sim \hbar^2\pi^2/(2m^*L^2)$, where ΔE is the energy spacing, $\hbar$ is the reduced Planck constant, m^* is the effective electron mass (~0.03 m_e), and L is the well width. This calculation yields an estimated spacing of ~0.1 meV—two orders of magnitude smaller than the observed 15-30 meV. Therefore, quantum confinement is unlikely to be responsible for the observed LDOS peaks.

3, **Alternative LDOS Explanation in DW region**

Pseudo-magnetic fields (PMFs) can also lead to discrete LDOS peaks via the formation of pseudo-Landau levels. However, the spatial width of the observed features excludes this possibility. In Bernal-stacked bilayer graphene, the Landau level energies follow the relation $E_N = \pm\hbar\omega_c\sqrt{N(N-1)}$, where $\omega_c = eB/m^*$, B is the magnetic field, m^* is the effective electron mass, and $N = 0, 1, 2, \ldots$ [6]. By fitting the LDOS spectrum, we extract a PMF of approximately 5.6 T for the 0.07°-twisted TBG. This field corresponds to a cyclotron radius of approximately 31 nm, which significantly exceeds the observed domain wall width (Fig. S2), indicating that a PMF cannot be sustained within the domain wall region. Therefore, the pseudo-Landau level mechanism can be excluded as the origin of the LDOS peaks.

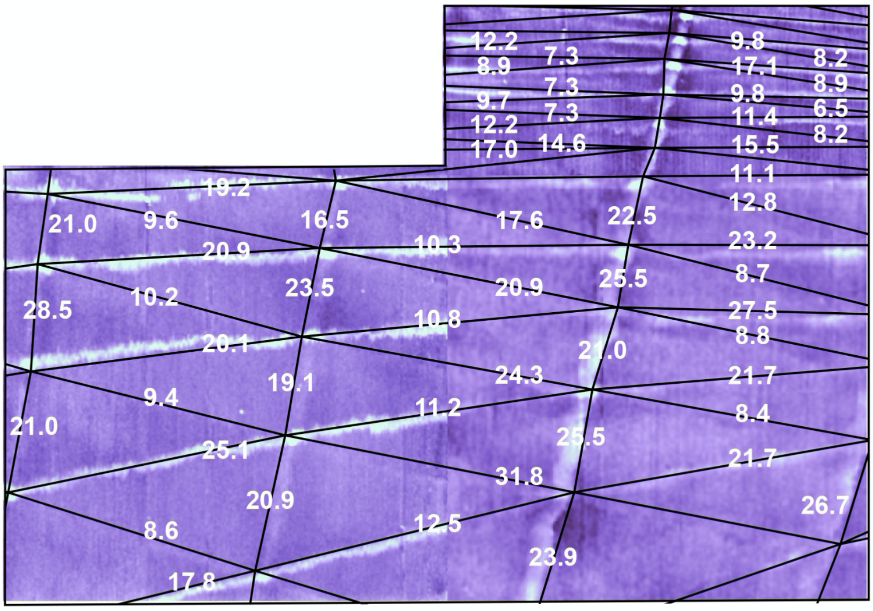

Figure S2. **Width of domain walls. Units are nanometers.** The DWs widths are extracted from line profiles taken perpendicular to the walls.

II. DETERMINATION OF TWIST ANGLE

In this section, we provide a general procedure to determine the strain profile of TBG for a given magnitude and direction of the real-space lattice vectors. We begin by choosing the lattice vectors of monolayer graphene as $\mathbf{a}_1 = a(1,0)$ and $\mathbf{a}_2 = a(\frac{1}{2}, \frac{\sqrt{3}}{2})$, where $a = 2.46$ Å is the lattice constant of graphene. For a twisted bilayer configuration with both twist and strain, the lattice vectors are given by

$$\boldsymbol{\alpha}_{i,\pm} = (1 + \mathcal{E}_{\pm})R\left(\pm\frac{\theta}{2}\right)\mathbf{a}_i, \tag{1}$$

where $R(\theta)$ is a rotation matrix and $\mathcal{E}_{\pm}$ is the strain tensor. $\boldsymbol{\alpha}_{i,\pm}$ are the lattice vectors for the top $(+)$ and bottom $(-)$ layers, respectively. For small deformations, the corresponding strained reciprocal lattice vectors are given by

$$\boldsymbol{\beta}_{i,\pm} = (1 - \mathcal{E}_{\pm})R\left(\pm\frac{\theta}{2}\right)\mathbf{b}_i, \tag{2}$$

where $\mathbf{b}_i$ is the reciprocal lattice vector of the non-strained lattice. In a general situation, the strain may differ between layers, this is $\mathcal{E}_+ \neq \mathcal{E}_-$. In the twisted system, assuming a commensurate lattice structure, the moiré reciprocal lattice vectors, $\mathbf{g}_i$, are given by the difference

$$\mathbf{g}_i = \boldsymbol{\beta}_{i,+} - \boldsymbol{\beta}_{i,-} \tag{3}$$

which can be expressed as $\mathbf{g}_i = \boldsymbol{T}\mathbf{b}_i$, with the transformation $\boldsymbol{T}$ defined as

$$\boldsymbol{T} = (1 - \mathcal{E}_-)R\left(-\frac{\theta}{2}\right) - (1 - \mathcal{E}_+)R\left(+\frac{\theta}{2}\right). \tag{4}$$

In an experiment, the moiré reciprocal vectors satisfy $\mathbf{g}_i \cdot \mathbf{L}_j = 2\pi\delta_{ij}$ where $\mathbf{L}_j$ are the primitive lattice vectors of the moiré cell. The strain tensor is symmetric, and it contains three independent components $\epsilon_{xx}, \epsilon_{yy}$ and ϵ_{xy}. In addition, $\mathbf{L}_1 = \{L_{1x}, L_{1y}\}$ and $\mathbf{L}_2 = \{L_{2x}, L_{2y}\}$. Therefore, by the orthogonality condition of the primitive and reciprocal lattice vectors we can write

$$(T_{xx}b_{ix} + T_{xy}b_{iy})L_{jx} + (T_{yx}b_{ix} + T_{yy}b_{iy})L_{jy} = 2\pi\delta_{ij} \tag{5}$$

Which after reorganizing terms can be written as a matrix equation of the form $\boldsymbol{MT} = \mathbf{v}$, with $\mathbf{v} = (2\pi, 2\pi, 0,0)$ and

$$\boldsymbol{T} = \begin{pmatrix} T_{xx} & T_{xy} \\ T_{yx} & T_{yy} \end{pmatrix} \tag{6}$$

The matrix is to be determined. Note that, the matrix elements of the strain tensor and the twist angle are contained in this matrix, this is, by given the primitive lattice vectors from the sample and by comparing with the electronic properties, the matrix T and hence the strain tensor and twist angle can be determined. For instance, according to the moiré periods in Fig. S3, which were obtained

from the STM topography, we can calculate the twist angle and strain in each moiré cell. Then, by comparing the electronic structures, we could find a pair of twist angle and strain that fits the best with experimental results [7]. The obtained shear strain distributions are plotted in Fig. S4.

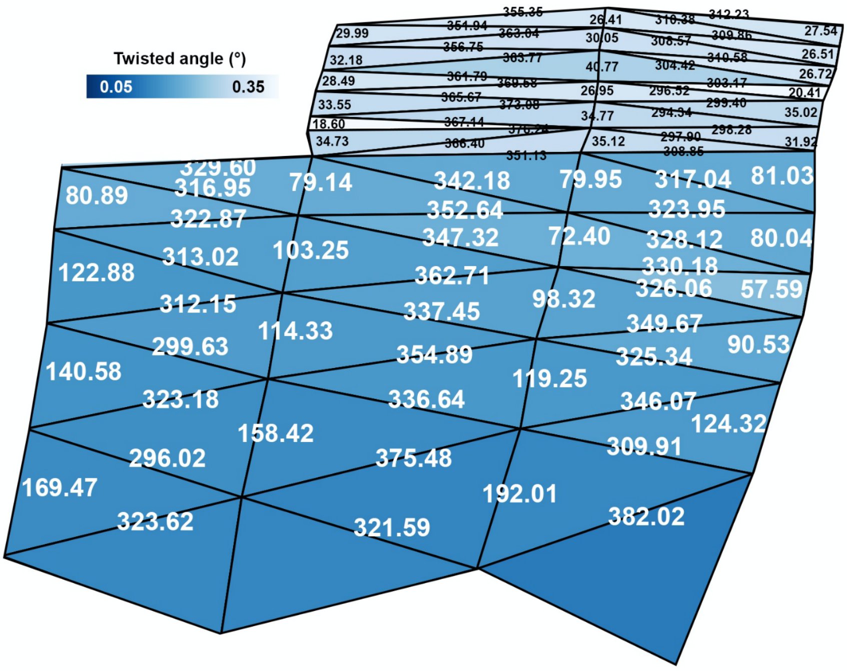

Figure S3. **Moiré periods of all triangles in TBG sample. Units are nanometers.**

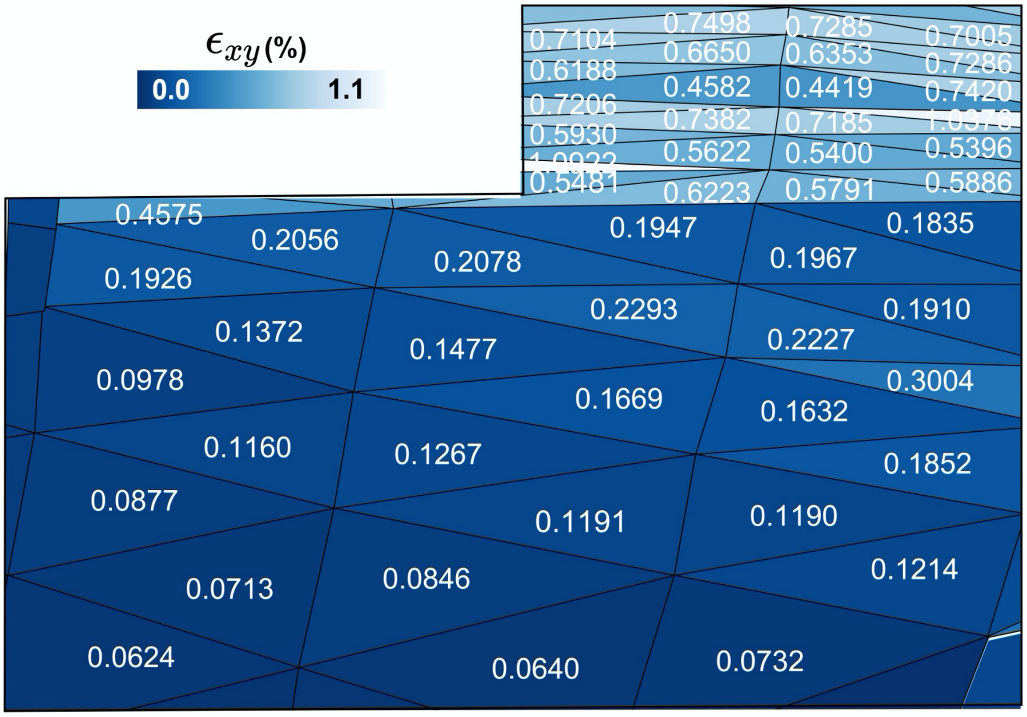

Figure S4. **Strain component** ϵ_{xy} **of all triangles in TBG sample.**

III. DIRECT SPECTROSCOPIC COMPARISON BETWEEN m-TBG and MATBG

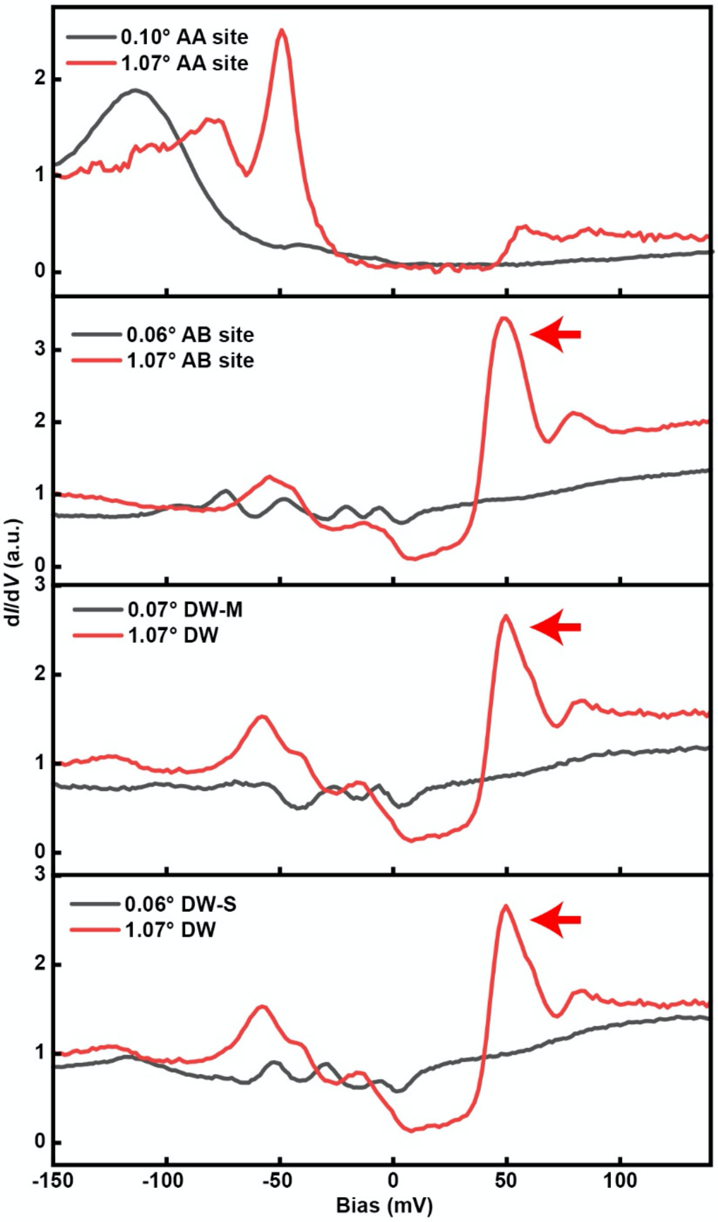

Figure S5. Direct spectroscopic comparison between m-TBG and MATBG. The overlaid dI/dV spectra compare magic-angle TBG (MATBG, red lines) and m-TBG (black lines) across various stacking regions (AA, AB, and DW), all measured on the same sample with identical setpoints and normalization. This comparison clearly show that the characteristic MATBG “remote band” signatures (marked by red arrows) are missing in m-TBG. Set point: V_b = -200 mV, I_t = 200 pA.

IV. TUNNELING SPECTRA OF DW-M

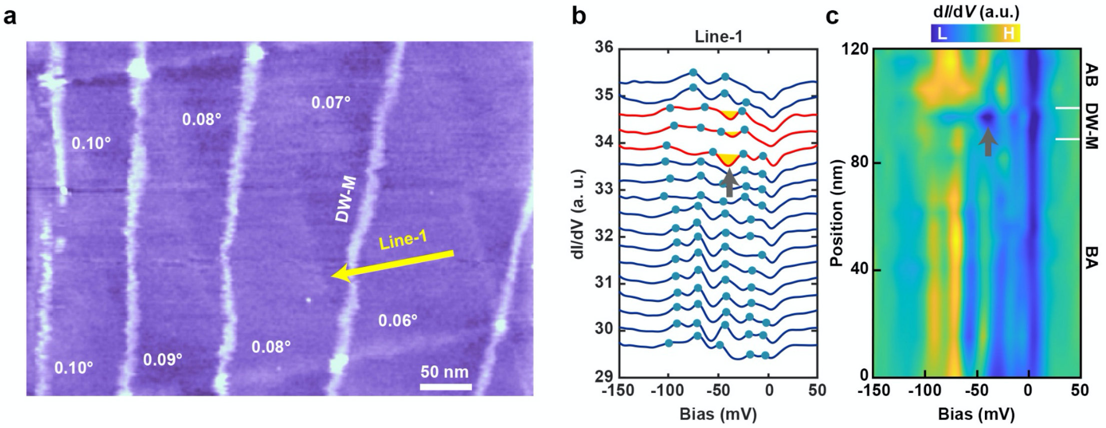

Figure S6. **Tunneling spectra of DW-M.** (**a**), STM topography (500 nm × 350 nm, V_b = -2000 mV, I_t = 20 pA) of TBG with twist angles ranging from 0.06° to 0.10°. In the picture, we name the DW with a bright topography as DW-M. (**b**), dI/dV spectra taken along the trajectories marked by Line-1 in (**a**), (**b**) dI/dV spectra taken along the Line-1 in (**a**), where red curves indicate DW-M spectra with a dip at -40 mV (gray arrow). The dI/dV spectra measured in the AB region has a dip at -60 mV. (**c**), Colormap of (**b**) highlights the dip at -40 mV in DW-M. Set point: (b-c), V_b = -200 mV, I_t = 200 pA.

V. REPRODUCIBILITY OF THE OBSERVATIONS

High-resolution measurements of individual domain walls [Figure S7(a)] reveal detailed electronic structure. The dI/dV colormap along Line-1 [Fig. S7(b)] shows a continuous spectral evolution across domain boundaries. Two distinct patterns are observed: (1) For DW-M transitions, the peaks shift symmetrically toward higher and lower energies, forming a characteristic “V”-shaped dispersion; in contrast, (2) DW-S transitions exhibit an asymmetric “Λ”-shaped pattern. These distinct behaviors suggest different coupling mechanisms between the domain walls and adjacent AB/BA regions, highlighting the complex interplay between atomic reconstruction and electronic structure in marginally twisted bilayer graphene.

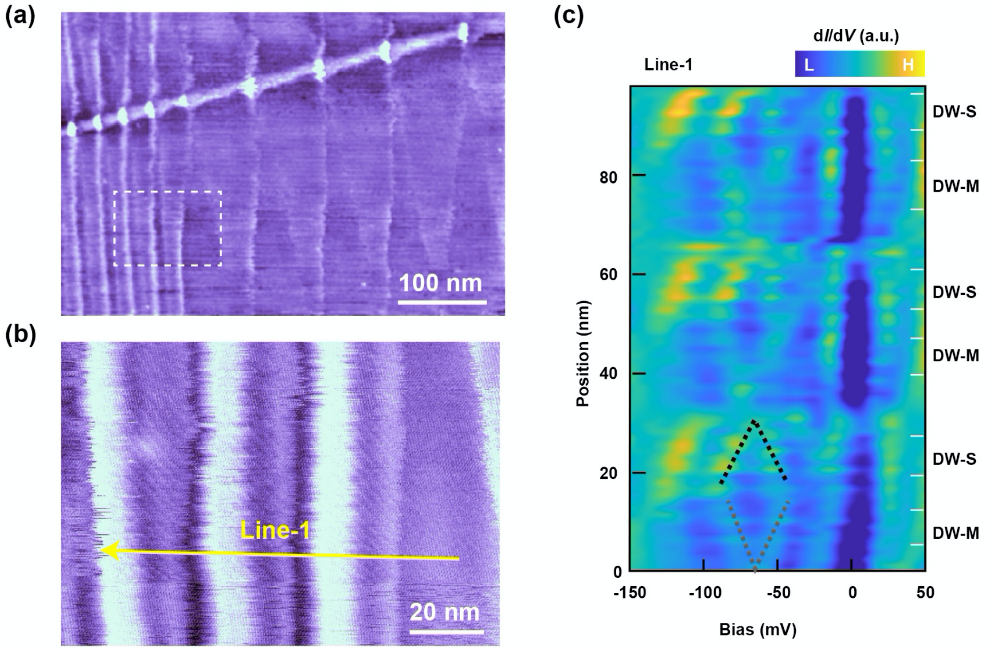

Figure S7. **Tunneling spectra of DW-M and DW-S in regions with rapidly changing DWs.** (**a**), STM topography (500 nm × 350 nm, V_b = -1000 mV, I_t = 20 pA) of TBG. (**b**), STM topography (120 nm × 80 nm, V_b = -200 mV, I_t = 20 pA) marked by white dashed square in (**a**). (**c**), Colormap of dI/dV spectra taken along the trajectories marked by Line-1 in (a). Set point: (b-g), V_b = -200 mV, I_t = 200 pA.

VI. TIGHT-BINDING CALCULATIONS OF ELECTRONIC PROPERTIES OF TBG WITH STRAIN

In this work, we use the full atomistic tight-binding (TB) method to study the electronic structures of TBG, considering the effect of strain and lattice relaxation. First, we use a general formalism to generate a commensurate structure. We assume the two graphene layers rotate in the plane by an angle θ with the rotation origin in the AA site. In a commensurate structure of TBG, the moiré lattice vector can be expressed as a function of those of the two graphene layers:

$$\begin{pmatrix} \mathbf{L}_1 \\ \mathbf{L}_2 \end{pmatrix} = \begin{pmatrix} i & j \\ k & l \end{pmatrix} \begin{pmatrix} \boldsymbol{\alpha}_{1,+} \\ \boldsymbol{\alpha}_{2,+} \end{pmatrix} = \begin{pmatrix} m & n \\ q & r \end{pmatrix} \begin{pmatrix} \boldsymbol{\alpha}_{1,-} \\ \boldsymbol{\alpha}_{2,-} \end{pmatrix} \tag{7}$$

where $\mathbf{L}_{1/2}$ is the lattice vector of the supercell, $\boldsymbol{\alpha}_{1,2,+}$ and $\boldsymbol{\alpha}_{1/2,-}$ are the lattice vector of the top and bottom graphene, respectively, i, j, k, l, m, n, q, r are eight integers. Then the lattice vectors of the top and bottom graphene layers can be related by a Park-Madden transformation matrix [8]:

$$\begin{pmatrix} \boldsymbol{\alpha}_{1,+} \\ \boldsymbol{\alpha}_{2,+} \end{pmatrix} = \frac{1}{il-jk} \begin{pmatrix} lm - jq & ln - jr \\ -km + iq & -kn + ir \end{pmatrix} \begin{pmatrix} \boldsymbol{\alpha}_{1,-} \\ \boldsymbol{\alpha}_{2,-} \end{pmatrix} \tag{8}$$

The commensurate supercell could also be expressed by the extended Wood's notation $(P_1R\theta_1, P_2R\theta_2)$, with $P_{1/2} = |\boldsymbol{\alpha}_{1,2,+}|/|\boldsymbol{\alpha}_{1/2,-}|$ the heterostrain amplitude, and $\theta_{1/2} = (\boldsymbol{\alpha}_{1,2,+}, \boldsymbol{\alpha}_{1/2,-})$ the rotation angle of the lattice vectors between the top and bottom layers. For a hexagonal lattice, the formalism of the extended Wood's notation is [9]:

$$\begin{pmatrix} \boldsymbol{\alpha}_{1,+} \\ \boldsymbol{\alpha}_{2,+} \end{pmatrix} = \begin{pmatrix} P_1(\cos\theta_1 + \frac{\sin\theta_1}{\sqrt{3}}) & \frac{2P_1}{\sqrt{3}}\sin\theta_1 \\ -\frac{2P_2}{\sqrt{3}}\sin\theta_2 & P_2(\cos\theta_2 - \frac{\theta_2}{\sqrt{3}}) \end{pmatrix} \begin{pmatrix} \boldsymbol{\alpha}_{1,-} \\ \boldsymbol{\alpha}_{2,-} \end{pmatrix} \tag{9}$$

The Eqs. (8) and (9) are identical. Therefore, once we know the twist angle and strain of the system, we could obtain a pair of eight integers, and then the commensurate structure will be known. For instance, if we choose the lattice vectors of the graphene as $\mathbf{a}_1 = a(\sqrt{3}/2, 1/2)$ and $\mathbf{a}_2 = a(\sqrt{3}/2, -1/2)$, for a TBG with twist angle $\theta = 0.35°$ only, the pair of integers is (189,-94,-95,189,189,-95,-94,189), and the number of atoms in one unit supercell is 107164. In the TBG with $\theta = 0.35°$ and a uniaxial heterostrain (only top layer is strained) $\varepsilon = 0.3\%$, the pair of eight integer is (152, -131, -20, 263, 152, -132, -19,263), and the number of atoms in one unit cell is 149648. For TBG with only $\theta = 0.2°$, the pair of integers is (331, -165, -166, 331, 331, -166, -165,331), and the number of atoms in one unit cell is 328684. For $\theta = 0.13°$, the pair of integers is (509, -254, -255, 509, 509, -255, -254,509) and one unit cell contains 777244 atoms. Figure S8 is the atomic structure of the TBG with $\theta = 0.35°$ and $\varepsilon = 0.3\%$.

After generating the commensurate structure, we introduce the effect of the lattice relaxation via the classical simulation package LAMMPS [10]. The intralayer and interlayer interactions are simulated with the long-range carbon bond-order potential [11] and Kolmogorov-Crespi [12] potentials. We assume the relaxed samples keep the same period of the rigid TBG. For TBG with tiny twist angles, the lattice relaxation changes significantly the atom positions from the rigid case, for instance, the AA sites is shrunk, the AB region is expanded to form a triangular pattern, and the

one-dimensional domain wall (DW) regions are generated, as shown in Figure S8. In Figure S8, we name the DWs labelled by the red and dark red rectangular as DW-M and DW-S, respectively. Figure S8 are more details of the changes in the AA, DW-M and DW-S.

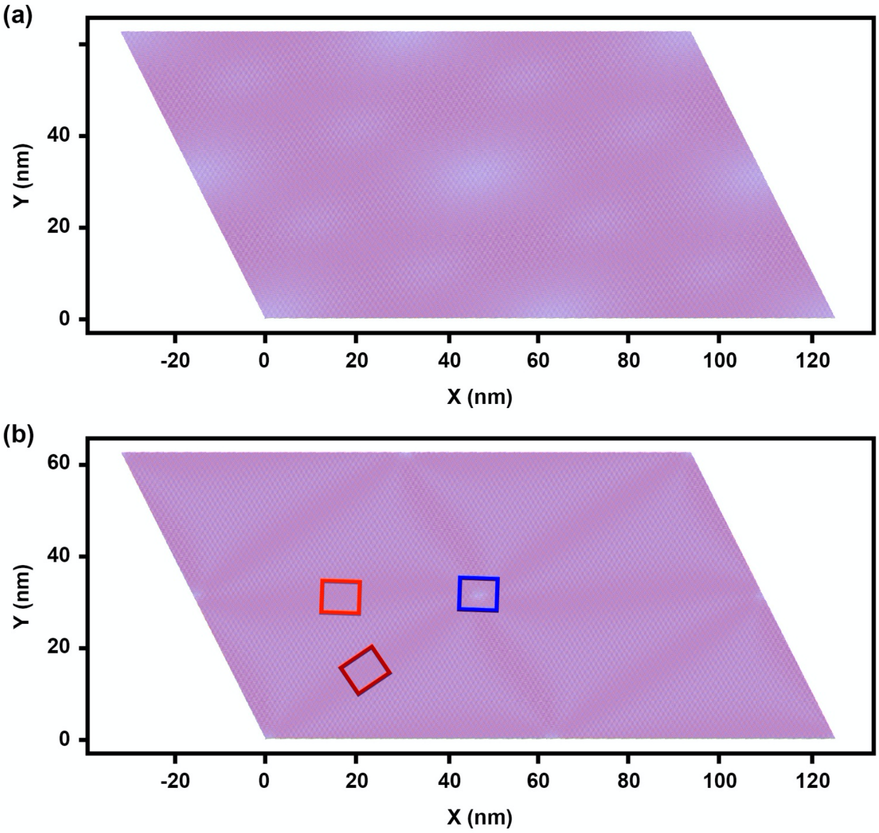

Figure S8. The 2×2 moiré cell. Generated atomic positions for the tight-binding calculations of TBG with $\theta = 0.35°$ and uniaxial heterostrain $\varepsilon = 0.3\%$ in the (**a**) rigid and (**b**) relaxed cases. The atoms in the top and bottom layers are in blue and red colors. The number of atoms in one unit cell is 149648. The AA, DW-M and DW-S regions are illustrated by blue, red and dark red rectangles, respectively.

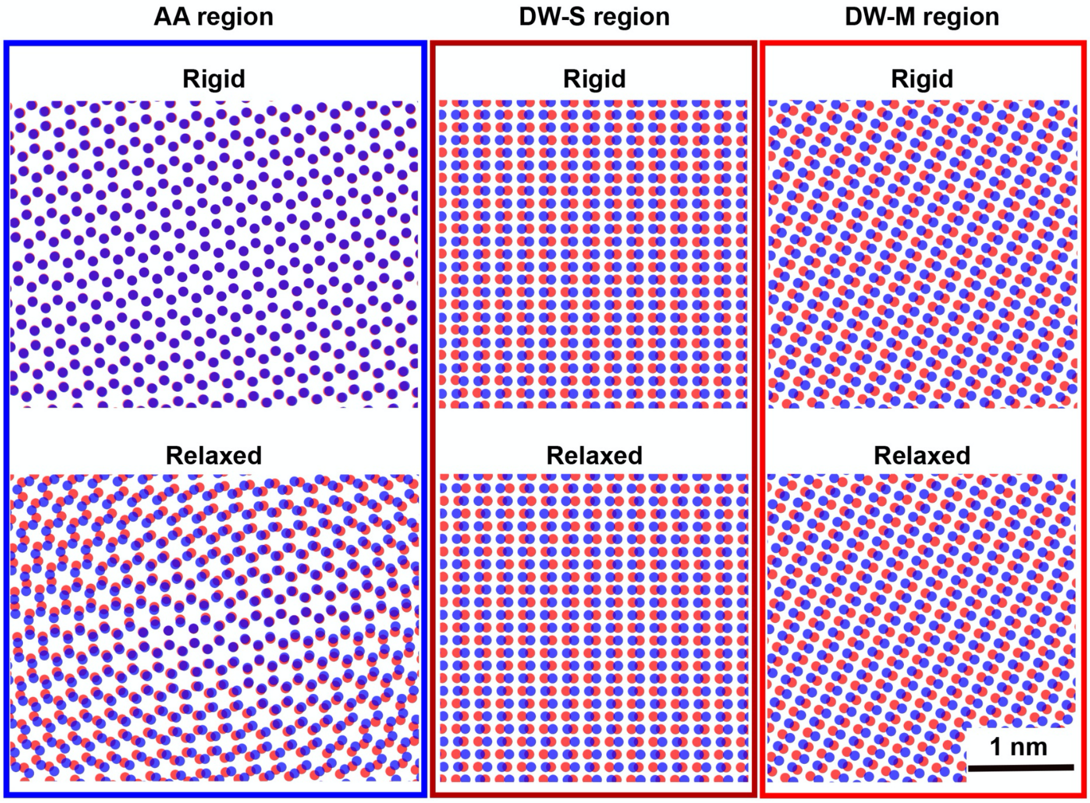

Figure S9. Local atomic structure before and after lattice relaxation. (Left panel) Near AA region, (middle panel) near DW-S region, and (right panel) near DW-M region.

We construct the full TB model by only consider the p_z orbital of the carbon atom [13]. The TB Hamiltonian is:

$$\mathrm{H} = \sum_i \epsilon_i |i\rangle\langle i| + \sum_{\langle i,j\rangle} t_{ij} |i\rangle\langle j| \quad (4)$$

where $|i\rangle$ is the p_z orbital located at $\mathbf{r}_i$, ϵ_i is the on-site energy, $\langle i,j\rangle$ is the sum over index i and j with $i \neq j$, t_{ij} is the hopping between two orbitals, which has a Slater-Koster formalism as:

$$t_{ij} = -(1-n^2)t_0 e^{q_\pi(1-r_{ij}/d)} + n^2 t_1 e^{q_\sigma(1-r_{ij}/h)} \quad (5)$$

where $r_{ij} = |\mathbf{r}_j - \mathbf{r}_i|$ is the distance between i and j orbitals, with n as the direction cosine along the direction $\mathbf{e}_z$ perpendicular to the graphene layer, t_0 and t_1 are hopping parameters, which are set to 2.8 eV and 0.44 eV, $d = 0.142$ nm and $h = 0.3349$ nm are the nearest in-plane and out-of-plane carbon-carbon distances, respectively, the parameters q_π and q_σ satisfy $\frac{q_\sigma}{h} = \frac{q_\pi}{d} = 2.218$ Å^{-1}. All the TB calculations are performed in the TBPLaS simulator [14].

VII. THE ATOMIC STRUCTURE OF DWS IN STRAINED TBG

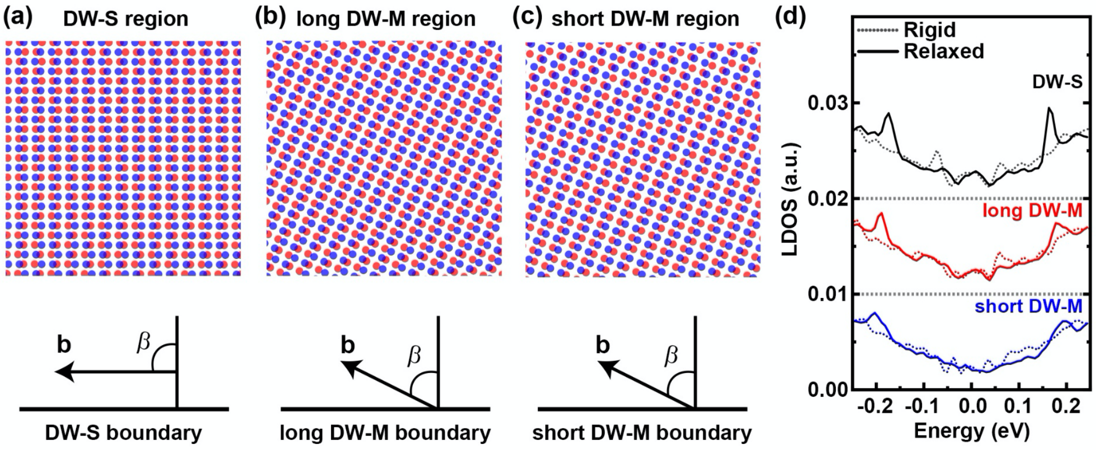

Figure S10. The atomic and electronic structures of the DWs in TBG with $\theta = 0.35°$ and uniaxial heterostrain $\varepsilon = 0.3\%$. (**a**) (Top panel) The atomic structure of the DW-S (dark red rectangle in Figure S8(b)), (Bottom panel) the scheme of the boundary of the DW-S and the Burger vector $\mathbf{b}$. (**b**) (Top panel) The atomic structure of the long DW-M (red rectangle in Figure S8(b)), (Bottom panel) the scheme of the boundary of the long DW-M and the Burger vector $\mathbf{b}$. (**c**) (Top panel) The atomic structure of the short DW-M, (Bottom panel) the scheme of the boundary of the short DW-M and the Burger vector $\mathbf{b}$. β is the angle between the Burger vector $\mathbf{b}$ and the normal to DWs. (**d**) The calculated LDOS of three DWs. The curves are relatively shifted to make the plot clear.

In the non-strain TBG with tiny angle, due to the lattice relaxation, the DWs generate a triangular network, and the DWs has a shear boundary, i.e. the angle between the Burger vector $\mathbf{b}$ (the shift of the unit cell across the DW) and the normal to the boundary aligned in armchair direction is $\beta = 90°$, as shown in Fig. S10(a). A tensile domain wall has $\beta = 0$. When a uniaxial strain is introduced in the system, for instance, a TBG with $\theta = 0.35°$ and uniaxial heterostrain $\varepsilon = 0.3\%$, different types of DWs are generated, as shown in Figure S10. The long DW-M and short DW-M are a combination of the shear and tensile DWs. This result is consistent with the previous theoretical work, which proposed a transition between the two different DWs under strain [15]. These different DWs show distinct LDOS, in particular, at the energy around -180 meV (the energy of the DW state induced by the lattice relaxation). The two DW-Ms have much weaker resonances around -180 meV than that of the DW-S.

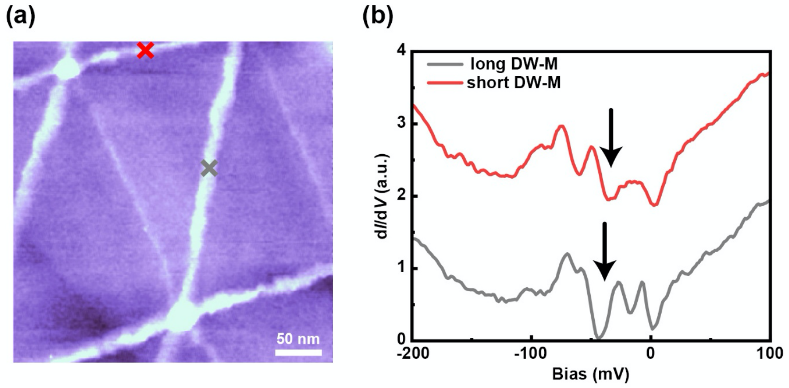

Figure S11. Direct spectroscopic comparison between long DW-M and short DW-M. (**a**) STM topography (350 nm × 350 nm, V_b = -2000 mV, I_t = 20 pA) of m-TBG region. (**b**) d*I*/d*V* spectra of long DW-M (grey cross) and short DW-M (red cross) from (a)**.** Black arrows denote the charge neutrality point. Both the long and short DW-M lack the peak of -120 meV. Short DW-M is vertically offset for clarity. Set point: V_b = -200 mV, I_t = 200 pA.

REFERENCES

1. Koshino M. Electronic transmission through AB-BA domain boundary in bilayer graphene. *Physical Review B* 2013; **88**: 115409.
2. San-Jose P, Gorbachev R V, Geim A K, *et al.* Stacking Boundaries and Transport in Bilayer Graphene. *Nano Letters* 2014; **14**: 2052-2057.
3. Timmel A, Mele E J. Dirac-Harper Theory for One-Dimensional Moiré Superlattices. *Physical Review Letters* 2020; **125**: 166803.
4. Alden J S, Tsen A W, Huang P Y, *et al.* Strain solitons and topological defects in bilayer graphene. *Proceedings of the National Academy of Sciences* 2013; **110**: 11256-11260.
5. Lin J, Fang W, Zhou W, *et al.* AC/AB Stacking Boundaries in Bilayer Graphene. *Nano Letters* 2013; **13**: 3262-3268.
6. Shi H, Zhan Z, Qi Z, *et al.* Large-area, periodic, and tunable intrinsic pseudo-magnetic fields in low-angle twisted bilayer graphene. *Nature Communications* 2020; **11**: 371.
7. Yu J, Jia G, Li Q, *et al.* Twist angle driven electronic structure evolution of twisted bilayer graphene. arXiv, 2024.
8. Artaud A, Magaud L, Le Quang T, *et al.* Universal classification of twisted, strained and sheared graphene moiré superlattices. *Scientific Reports* 2016; **6**: 25670.
9. Hermann K. Periodic overlayers and moiré patterns: theoretical studies of geometric properties. *Journal of Physics: Condensed Matter* 2012; **24**: 314210.
10. Fast Parallel Algorithms for Short-Range Molecular Dynamics. *Journal of Computational Physics* 1995; **117**: 1-19.
11. Los J H, Ghiringhelli L M, Meijer E J, *et al.* Improved long-range reactive bond-order potential for carbon. I. Construction. *Physical Review B* 2005; **72**: 214102.
12. Kolmogorov A N, Crespi V H. Registry-dependent interlayer potential for graphitic systems. *Physical Review B* 2005; **71**: 235415.
13. Trambly de Laissardière G, Mayou D, Magaud L. Numerical studies of confined states in rotated bilayers of graphene. *Physical Review B* 2012; **86**: 125413.
14. TBPLaS: A tight-binding package for large-scale simulation. *Computer Physics Communications* 2023; **285**: 108632.
15. Lebedeva I V, Popov A M. Two Phases with Different Domain Wall Networks and a Reentrant Phase Transition in Bilayer Graphene under Strain. *Physical Review Letters* 2020; **124**: 116101.
